# Evaluation of corneal epithelial thickness mapping using anterior segment OCT in children with vernal keratoconjunctivitis

**DOI:** 10.1007/s10792-022-02596-9

**Published:** 2022-12-03

**Authors:** Maha Alsayed Albadawi, Ghada Azab Nassar, Heba A El Gendy, Dalia AbdelFattah Ghalwash

**Affiliations:** Faculty of Medicine, Cairo University, Manial, Cairo, 1107 Egypt

**Keywords:** Corneal epithelial thickness mapping, Anterior segment OCT, Vernal keratoconjunctivitis, Allergic conjunctivitis

## Abstract

**Purpose:**

To evaluate the corneal epithelial thickness by anterior segment OCT in children with vernal keratoconjunctivitis (VKC) compared to normal participants.

**Patient and methods:**

A cross-sectional case control observational study was conducted on children aged from 5 to 18 years with VKC. The study included 142 eyes divided into two groups: 71 eyes of VKC group and 71 eyes of age matched control group. Anterior segment OCT epithelial mapping for the central 5-mm was done to evaluate epithelial thickness-based variables.

**Results:**

Corneal epithelial thickness mapping showed significant superior thinning (51.07 ± 4.11) μm in VKC group compared to controls (52.54 ± 2.01) μm (*p* = 0.008), a decreased MIN epithelial thickness (45.99 ± 6.52) μm compared to controls (50.11 ± 1.91) μm (*p* < 0.001) and more negative (MIN–MAX) value (− 11.77 ± 9.38) indicating focal epithelial thinning compared to (− 5.80 ± 1.88) in controls (*p* = 0.001). In palpebral VKC, MIN epithelial thickness showed significant thinning (48. 38 ± 3.45) μm compared to controls (50.11 ± 1.91) μm (*p* = 0.001). Eyes with mixed VKC showed superior epithelial thinning (50.70 ± 4.59) μm compared to (52.54 ± 2.01) μm in controls (*p* = 0.025) and significant decreased MIN epithelial thickness (43.78 ± 7.83) μm compared to (50.11 ± 1.91) μm in control group.

**Conclusions:**

VKC is a disease primarily affecting the corneal epithelium. Corneal epithelial thickness mapping may be considered to assess the integrity of the ocular surface in eyes with VKC, and to detect corneal epithelial changes. Disease phenotype may influence the corneal epithelial changes, and the disease duration is another factor influencing these changes.

## Introduction

Vernal keratoconjunctivitis (VKC) is a chronic, recurrent bilateral allergic inflammation of conjunctiva and cornea that tend to occur in children and young adults. Despite the noted data that most types of allergic conjunctivitis do not affect vision, VKC may cause damage to the cornea with subsequent vision threatening complications [1].

VKC usually presents in children less than 10 years of age with a peak incidence between the ages of 11 and 13 years old, although 10% of VKC patients are older than 20 years old at the time of onset. Males are affected twice as often as females, whereas many patients with VKC have symptoms that are exacerbated in the spring, possibly due to the increase in pollen count [2].

Clinical and immune-histochemical studies suggest that IgE-dependent (type I allergic) and IgE-independent (type IV allergic) mechanisms are involved in the immunopathogenesis of VKC, in which various inflammatory cells, including different T cell subpopulations, play an active role via a cascade of chemical mediators [3].

Published studies reported variable rates of associations between VKC and the development of keratoconus (KC), whereas KC was detected at incidences of 26.8%, 8.5%, and 18.3% in patients with VKC using quantitative videokeratography maps, slit lamp biomicroscopy, and keratometry, respectively, with the increased incidence of keratoconus was associated with male gender, long-standing disease, mixed and palpebral forms, and advanced corneal lesions. Others reported lower incidences (0.77–1.33%) [4, 5].

Topographic corneal changes have been recorded in eyes with VKC, with keratoconus-like topographic changes. A trend of superior corneal steepening (‘superior keratoconus’) was also found [6–8].

The epithelial thickness profile maps have been proposed as an adjunctive tool to improve the sensitivity and specificity of keratoconus screening. Thus, in eyes with early keratoconus, epithelial compensation can mask the presence of an underlying cone on front surface topography, i.e., a diagnosis of keratoconus might be missed. Moreover, the presence of specific epithelial patterns, i.e., Doughnut pattern has been reported as an indicator for the presence of an underlying stromal cone [9, 10].

We aimed to study the corneal epithelial thickness mapping among children with VKC aged between 5 and 18 years, for the possibility of the prediction of early changes and the proposal of a new protocol for the management and follow-up in pediatric VKC patients.

## Patients and methods

A cross-sectional case control observational study was conducted on 71 eyes of children with VKC (34 eyes of palpebral type (47.9%) and 37 eyes of mixed type (52.1%) who attended the outpatient clinics at Kasr Al Ainy hospitals, Faculty of medicine, Cairo University for the period between February 2019 to June 2019, and those with a recorded medical history of VKC in the registry system were recruited for participation in the study. However, most of our patients were recruited in March and April (about 70% of the study subjects). All the obtained data were compared to the data retrieved from 71 eyes of age matched normal control group.

The study protocol was approved by the scientific committee of Cairo University. An informed consent was obtained according to the tenets of Declaration of Helsinki for all study participants after explaining the nature of the study. Consent was given by the parent/guardian.

All participants aged 5–18 years with a history of VKC (itching, mucous discharge, photophobia, foreign body sensation), and otherwise, a clinically normal cornea with no clinically detected corneal pathology and/ or signs of dry eye were included.

Participants who present with vernal keratoconjunctivitis were included after clinical improvement and stoppage of topical medications especially corticosteroids for at least 4 weeks period duration to avoid any confounding factors that may affect the epithelial map imaging. A multidisciplinary team was available with collaboration between ophthalmologists, immunologists and pediatricians to manage possible complications. However, no complex cases were encountered in the study.

### Methodology

- All the study participants were subjected to detailed relevant history-taking. This included complete personal history, complaint, and its duration; specifically, patients were asked about the presence or absence of redness, discharge, itching and photophobia and its duration, family history of similar conditions of allergy and consanguinity, presence of associated atopy (eczema, allergic rhinitis, or allergic asthma), and history of previous treatment.
- Slit lamp examination was performed on each patient to diagnose allergic conjunctivitis according to the type of allergy into seasonal/perennial allergic conjunctivitis and vernal/atopic keratoconjunctivitis. The latter classified into palpebral, limbal/corneal, and mixed. Allergic conjunctivitis different types were diagnosed taking into consideration conjunctival signs, corneal and limbal involvement.

The clinical classification of VKC into palpebral, limbal/corneal and mixed was established by Emmert in 1888 [11]. These types are defined as:

1. Limbal VKC: is distinguished by the development of papillae at the limbus without giant papilla formation on the tarsal conjunctiva.
2. The palpebral type: is distinguished by giant papillae on the upper tarsal conjunctiva without limbal papillae.
3. Mixed type: is showing both giant papillae and limbal papillae.
  Binocular indirect ophthalmoscope for fundus examination, and measurement of intraocular pressure (Goldmann’s applanation/Perkin’s tonometer).
  Measurement of best corrected visual acuity (BCVA) (Snellen chart) and conversion to logarithm of minimum angle resolvable (Log MAR).
  For all patients fulfilling the inclusion criteria, anterior segment OCT scans were performed on both eyes using Optovue RTVue model RT 100, Optovue, Inc., Fremont, CA. All anterior segment OCT scans were performed with the patient instructed to blink several times before the procedure, and a drop of lubricant eye drops was instilled. One scan per eye was obtained for each patient. Every examination was critically reviewed for quality of images, alignment, and measurement coverage; uncooperative young patients with poor quality imaging were excluded. In the case of poor quality (e.g., from excessive movement or from poor quality tear film), a new scan was repeated, and in further failure, the patient was excluded from the study. Patients with limbal stem cell deficiency (LSCD) were excluded from the study. LSCD was diagnosed by the presence of epithelial breakdown, persistent epithelial defects, loss of palisades of Vogt and perilimbal vascular arcades. Conjunctivalization, corneal neovascularization and corneal scarring were excluded from the study.
  Interpretation of epithelial thickness maps and variables.

A 6-mm-diameter epithelial thickness map is generated by interpolating epithelial thickness profiles calculated from each meridian. Only the central 5-mm-diameter map is used for calculating epithelial thickness-based variables.

**Fig. 1:**
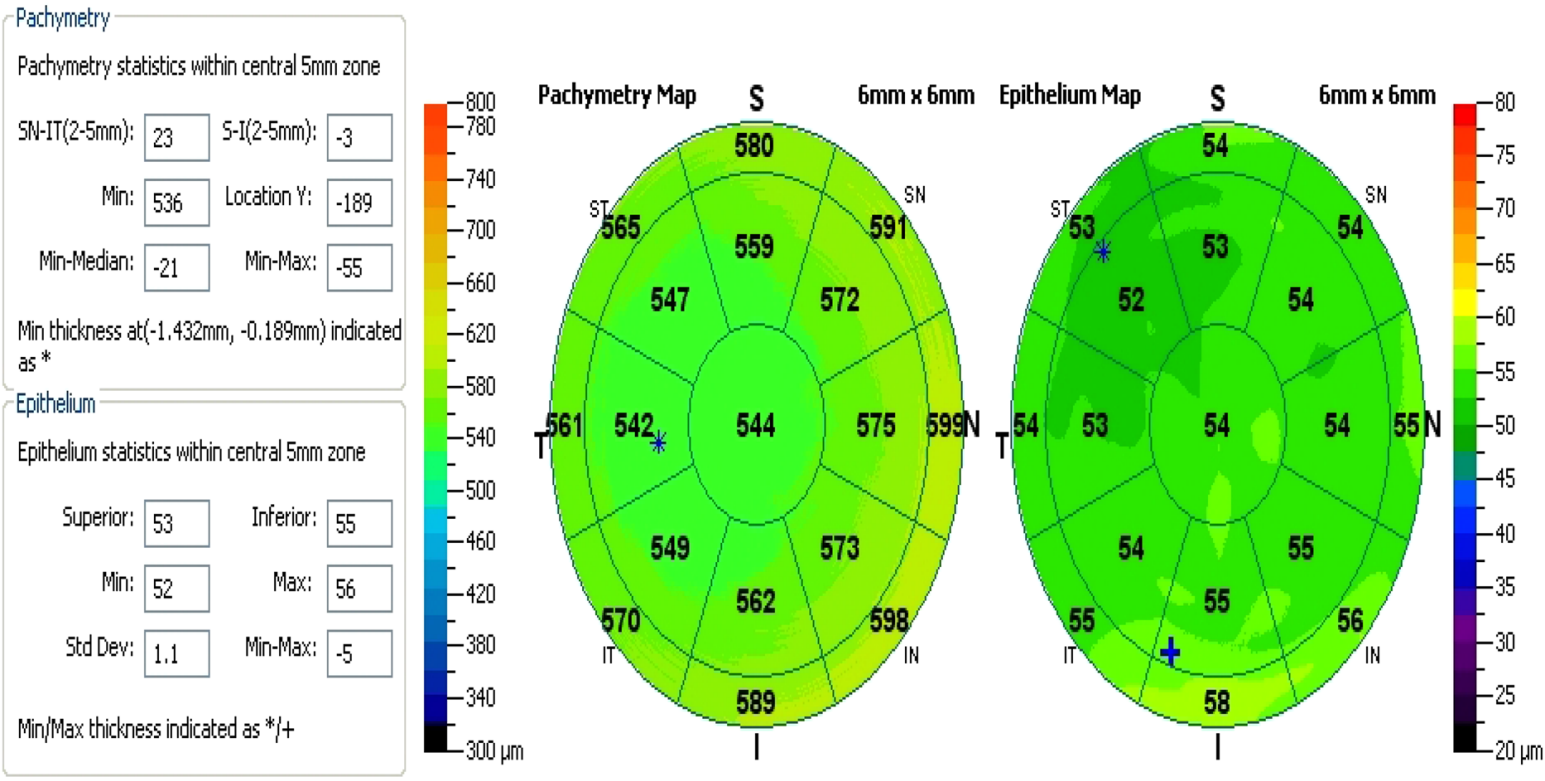
Epithelial and pachymetry mapping in a normal control eye

The epithelial thickness map is divided into 3 zones by diameter and hemispheres: central 2 mm, superior 2–5 mm, and inferior 2–5 mm. The average epithelial thicknesses of central, superior, and inferior zones are calculated (Fig. 1).

The minimum and maximum epithelial thicknesses are recorded, and the epithelial focal thinning is calculated as the difference between them (minimum − maximum, or MIN − MAX). Superior minus inferior asymmetry (superior − inferior, or S − I) also is calculated by taking the difference between the average epithelial thicknesses of the superior and inferior zones.

### Statistical analysis

Data were coded and entered using the statistical package SPSS version 25. Data were summarized using mean, standard deviation, median, minimum, and maximum for quantitative variables and frequencies (number of cases) and relative frequencies (percentages) for categorical variables. Comparisons between groups were done using unpaired t test in normally distributed quantitative variables, while nonparametric Mann–Whitney test was used for non-normally distributed quantitative variables*.* For comparing categorical data, Chi-square (*χ*^2^) test was performed. Exact test was used instead when the expected frequency is less than 5*.* Correlations between quantitative variables were done using Spearman correlation coefficient. *p *values less than 0.05 were considered as statistically significant.

## Results

### Demographic data

In the current study, there were statistically insignificant difference between both group regarding the age and sex. The mean age was 8.15 ± 2.41 years in VKC group compared to 7.99 ± 1.31 years in control group (*p* = 0.605), also the male to female ratio was 64.8%:35.2% in VKC group compared to 56.3%:43.7% in control group (*p* = 0.303) (Table1). Regarding consanguinity, it was positive in 28.2% of VKC patients, and a positive family history of VKC was recorded in 33.8% of VKC group. The mean disease duration in our study was 2.86 ± 2.1 years.

**Table 1:** The demographic data of the VKC group compared to the control group

	VKC (*n* = 71)	Control (*n* = 71)	*p* value
	Mean ± SD	Mean ± SD	
Age (year)	8.15 ± 2.41	7.99 ± 1.31	0.605

		Number	%	Number	%	
*Sex*	Male	46	64.8	40	56.3	0.303
	Female	25	35.2	31	43.7	

### Clinical data

This study shows that BCVA was statistically decreased 0.28 ± 1.09 Log MAR in VKC group compared to 0.08 ± 0.18 Log MAR in control group (*p* = 0.014). Moreover, there was a significant difference between the VKC group and the control group regarding the spherical equivalent which was 0.87 ± 0.92 in VKC group compared to 0.62 ± 1.05 in control group ( *p* = 0.001). While there was no significant difference in the astigmatism in both groups, it was 1.00 ± 0.90 in VKC group compared to 1.23 ± 0.61 in control group (*p* = 0.052). Intraocular pressure showed a significant difference in both groups with a mean value of 12.66 ± 2.38 mmHg in VKC group compared to 10.25 ± 1.24 mmHg in control group (*p* < 0.001) (Table 2).

**Table 2:** Comparison between VKC and control groups regarding BCVA, spherical equivalent, astigmatism, and intraocular pressure

	VKC group (*n* = 71)	Control group (*n* = 71)	*p* value
	Mean ± SD	Mean ± SD	
BCVA (Log MAR)	0.28** ± **1.09	0.08** ± **0.18	0.014*
spherical equivalent	0.87** ± **0.92	0.62** ± **1.05	0.001*
Astigmatism	1.00** ± **0.90	1.23** ± **0.61	0.052
IOP (mmHg)	12.66** ± **2.38	10.25** ± **1.24	< 0.001*

*BCVA* best corrected visual acuity, *IOP* intraocular pressure

*Significant

### Parameters of epithelial thickness map

**Fig. 2:**
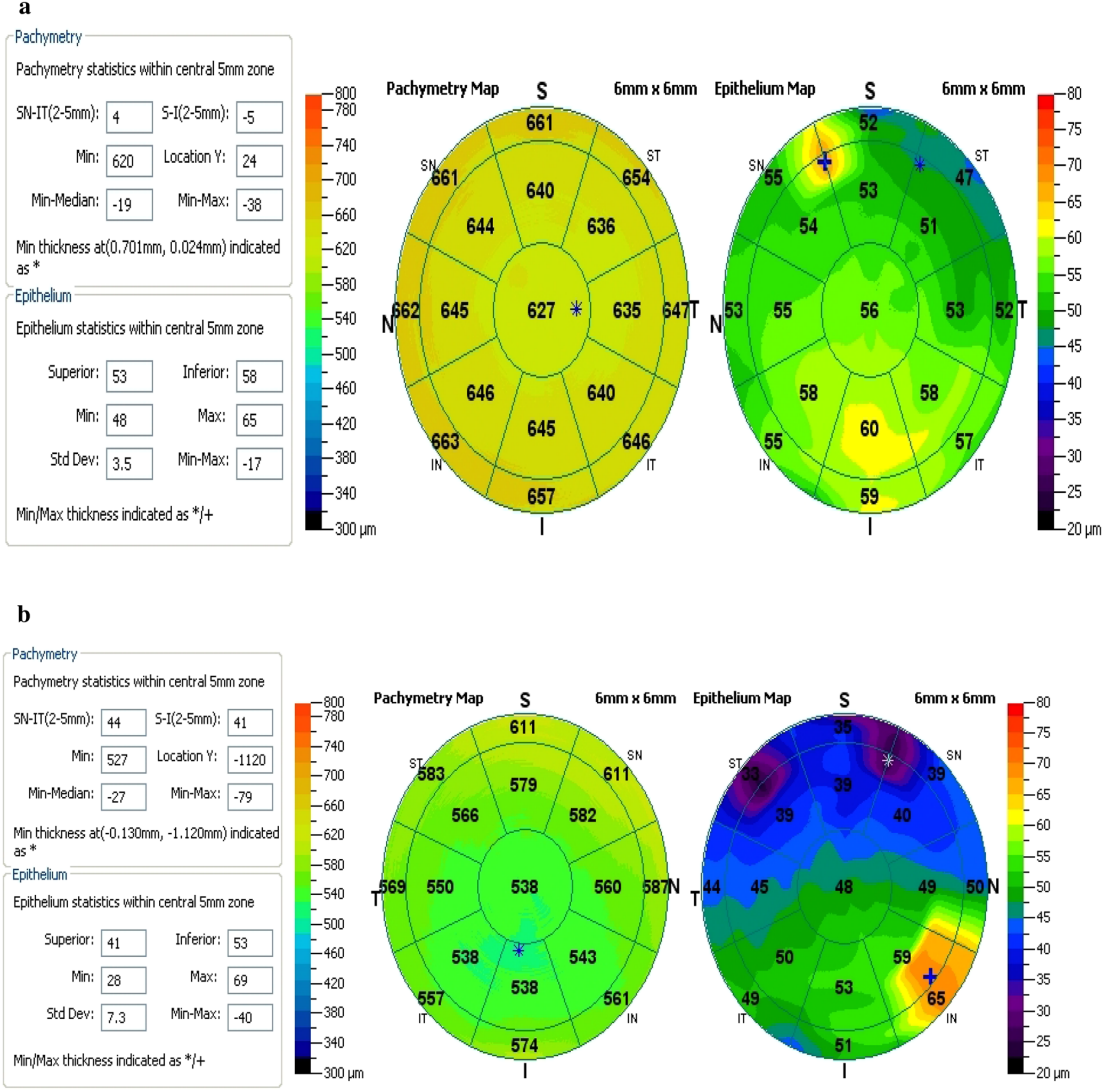
Epithelial and pachymetry mapping in a VKC eye: **a** (upper) palpebral type, **b** (bottom) mixed type, demonstrating superior thinning

This study showed that children in VKC group had significant superior thinning in the corneal epithelial thickness map about (51.07 ± 4.11) μm compared to control group which was (52.54 ± 2.01) μm (*p* = 0.008). Moreover, there was a highly significant decrease in the MIN epithelial thickness (45.99 ± 6.52) μm among children in VKC group than in the control group (50.11 ± 1.91) μm

(*p* < 0.001) and more negative value (MIN–MAX) in VKC group (− 11.77 ± 9.38) indicating focal epithelial thinning compared to (− 5.8 ± 1.82) in control group (*p* = 0.001). However, there was no significant statistical difference between both groups regarding other parameters (Fig. 2a, b; Table 3).

**Table 3:** Epithelial thickness map parameters in VKC and Control groups in μm

	VKC group (*n* = 71)	Control group (*n* = 71)	*p* value
	Mean ± SD	Mean ± SD	
CET	53.01 ± 3.75	53.55 ± 1.39	0.263
SUP	51.07 ± 4.11	52.54 ± 2.01	0.008*
INF	52.93 ± 3.84	53.30 ± 1.86	0.472
MIN	45.99 ± 6.52	50.11 ± 1.91	< 0.001*
MAX	57.76 ± 6.20	55.92 ± 2.32	0.021
MIN−MAX	− 11.77 ± 9.38	− 5.80 ± 1.88	< 0.001*
SUP−INF	− 1.86 ± 2.87	− 0.76 ± 1.20	0.008*
Epithelial thickness at the area of minimal corneal thickness	52.25 ± 4.22	53.08 ± 1.93	0.135

*CET* central epithelial thickness, *SUP* superior, *INF* inferior, *MIN* minimum, *MAX* maximum, *MIN−MAX*, minimum minus maximum, *SUP−INF*, superior minus inferior

*Significant

Furthermore, correlating the epithelial thickness mapping parameters with the disease duration, revealed that the minimum epithelial thickness significantly decreased with increase in disease duration (*r* = 0.339) (*p* = 0.004) and the MIN − MAX value became more negative (*r* = 0.259) (*p* = 0.029), whereas other parameters showed no significant correlation (Table 4).

**Table 4:** Correlation of epithelial mapping parameters, stromal and total corneal thickness with disease duration

Parameter	Correlation coefficient (*r*)	*p* value
CET	0.011	0.928
SUP	0.068	0.573
INF	0.054	0.653
MIN	0.339	0.004*
MAX	0.167	0.163
MIN−MAX	0.259	0.029*
SUP−INF	0.028	0.814
CCT	0.090	0.455
MIN (Pachymetry)	0.098	0.415
Stromal thickness at the area of maximum epithelial thickness	0.152	0.205
Stromal thickness at the thinnest corneal point	0.122	0.310

*CET* central epithelial thickness, *SUP* superior, *INF* inferior, *MIN* minimum, *MAX* maximum, *MIN*−*MAX* minimum minus maximum, *SUP*−*INF* superior minus inferior, *CCT* central corneal thickness

*Significant

The pachymetry map parameters in both VKC and control group showed no significant statistical difference as shown in Table 5.

**Table 5:** Pachymetry map parameters in VKC and control groups in μm

	VKC group (*n* = 71)	Control group (*n* = 71)	*p* value
	Mean ± SD	Mean ± SD	
CCT	535.35 ± 30.51	539.65 ± 23.45	0.349
MIN (total thickness)	523.48 ± 36.73	534.65 ± 27.03	0.041*

*CCT central corneal thickness, *MIN* minimum

In a trial to demonstrate how the clinical phenotype of disease presentation can influence the corneal epithelial parameters, we further sub-grouped the VKC patients into two; palpebral and mixed VKC subgroups and again, we compared the parameters of epithelial thickness map in each group with the control group as well as with each other.

In mixed VKC group, there was superior epithelial thinning with mean ± SD (50.70 ± 4.59) μm compared to (52.54 ± 2.01) μm in control group (*p* = 0.025), and MIN epithelial thickness decreased significantly with mean ± SD (43.78 ± 7.83) μm compared to (50.11 ± 1.91) μm in control group (*p* < 0.001). Also, MAX epithelial thickness increased significantly in mixed VKC group with mean ± SD (59.57 ± 7.00) μm compared to (55.92 ± 2.32) μm in control group (*p* = 0.004); as shown in Fig. 2, and MIN–MAX epithelial thickness was more −ve with mean ± SD (− 15.78 ± 11.06) compared to (− 5.80 ± 1.88) in control group (*p* < 0.001). Moreover, SUP − INF epithelial thickness was higher in mixed VKC group with mean ± SD (− 2.54 ± 3.32) compared to (− 0.76 ± 1.20) in control group which indicated asymmetry (*p* = 0.003) as shown in Table 6.

**Table 6:** Epithelial map parameters in mixed VKC and Control groups

	Mixed type (*n* = 37)	Control group (*n* = 71)	*p* value
	Mean ± SD	Mean ± SD	
CET	53.38 ± 3.78	53.55 ± 1.39	0.792
SUP	50.70 ± 4.59	52.54 ± 2.01	0.025*
INF	53.24 ± 4.09	53.30 ± 1.86	0.941
MIN	43.78 ± 7.83	50.11 ± 1.91	< 0.001*
MAX	59.57 ± 7.00	55.92 ± 2.32	0.004*
MIN−MAX	− 15.78 ± 11.06	− 5.80 ± 1.88	< 0.001*
SUP−INF	− 2.54 ± 3.32	− 0.76 ± 1.20	0.003*
Epithelial thickness at the area of minimal corneal thickness	53.00 ± 4.43	53.08 ± 1.93	0.912

*CET* central epithelial thickness, *SUP* superior, *INF* inferior, *MIN* minimum, *MAX* maximum, *MIN−MAX*, minimum minus maximum, *SUP−INF*, superior minus inferior

*Significant

In palpebral VKC group, only MIN epithelial thickness showed more thinning with a mean ± SD (48. 38 ± 3.45) μm compared to (50.11 ± 1.91) μm in control group (*p* = 0.001), whereas other epithelial thickness map parameters showed no significant statistical difference.

Epithelial thickness map analysis comparing parameters in the study group according to the initial clinical presentation showed a significant difference in MIN epithelial thickness parameters between eyes presented with mixed type VKC, and those presented with palpebral form with a mean ± SD were (43.78 ± 7.83) μm and (48.38 ± 3.45) μm, respectively (*p* = 0.002), and in mixed group MAX epithelial thickness mean ± SD was (59.57 ± 7.00) compared to (55.79 ± 4.51) μm in palpebral group *(p* = 0.008), also MIN–MAX mean ± SD of mixed group was (− 15.78 ± 11.06) compared to(− 7.41 ± 3.92) in palpebral group *(p* ≤ 0.001) (Table 7)*.*

**Table 7:** Epithelial thickness map parameters in mixed and Palpebral groups

	Mixed type (52.1%)	Palpebral type (47.9%)	*p* value
	Mean ± SD	Mean ± SD	
CET	53.38 ± 3.78	52.62 ± 3.73	0.397
SUP	50.70 ± 4.59	51.47 ± 3.54	0.436
INF	53.24 ± 4.09	52.59 ± 3.59	0.477
MIN	43.78 ± 7.83	48.38 ± 3.45	0.002*
MAX	59.57 ± 7.00	55.79 ± 4.51	0.008*
MIN−MAX	− 15.78 ± 11.06	− 7.41 ± 3.92	< 0.001*
SUP−INF	− 2.54 ± 3.32	− 1.12 ± 2.09	0.107
Epithelial thickness at the area of minimal corneal thickness	53.00 ± 4.43	51.44 ± 3.88	0.121

*CET* central epithelial thickness, *SUP* superior, *INF* inferior, *MIN* minimum, *MAX* maximum, *MIN−MAX*, minimum minus maximum, *SUP−INF*, superior minus inferior

*Significant

For the AS-OCT pachymetry map, the total corneal thickness and stromal thickness in different areas showed no significant statistical difference between mixed and palpebral VKC groups in all parameters, apart from a significant less stromal thickness at the area of maximum epithelial thickness. The mean ± SD was (486.78 ± 30.88) μm in mixed group compared to (502.94 ± 34.70) μm in palpebral group *(p* = 0.042) (Table 8).

**Table 8:** Stromal thickness in different areas in μm in mixed and palpebral groups

	Mixed type	Palpebral type	*p* value
	Mean ± SD	Mean ± SD	
Stromal thickness at the area of maximum epithelial thickness	486.78 ± 30.88	502.94 ± 34.7	0.042*
Stromal thickness in the thinnest corneal point	469.59	473.00	0.700

*Significant

## Discussion

Vernal keratoconjunctivitis (VKC) is a chronic recurrent bilateral allergic disease affecting both conjunctiva and cornea and usually, occurs in children and young adults. Also characterized by seasonal exacerbation and predominantly presents in hot and dry climates. This type of allergic inflammation may cause damage to the cornea and induce serious visual changes [1].

The present study was conducted to evaluate AS-OCT epithelial thickness map possible changes in eyes with VKC among the pediatric age group population as compared to healthy control group.

The age ranged from 5 to 18 years with a mean age of 8.15 ± 2.41 in the VKC group. That was concise with different reported studies on VKC patients addressing nearly the same age group [2, 6, 12].

To our knowledge, the current study is the first study to evaluate corneal epithelial thickness changes using AS-OCT in VKC patients, with no similar studies were recorded in literature.

The present study showed a statistically significant superior corneal epithelial thinning with an overall decrease in minimal epithelial thickness values that were recorded in VKC eyes compared to control group, whereas, other studies found superior corneal steepening “superior keratoconus” (a negative value for the I–S index using videokeratography) in the VKC group [6–8].

Moreover, higher negative values for MAX − MIN parameter of statistical significance were also recorded denoting significant focal thinning in the VKC eyes as compared to control group eyes.

On the other hand, studying the pachymetry maps in both groups revealed no significant difference between both groups regarding the CCT& MIN(total thickness) with normal recorded mean values in both groups; despite the relatively lower values among VKC eyes, denoting that VKC is mostly a disease affecting the corneal epithelium with no or late effect on the stromal tissue.

In the present study, it was found that the MIN values decreased significantly with the increase in disease duration; with the MIN − MAX values becoming more negative with the disease progression. We assumed that the disease duration plays an important role in inducing changes to corneal epithelial thickness map value.

Further subdividing VKC group into mixed and palpebral groups and comparing each group individually with the control group revealed that the epithelial changes were more evident in mixed type VKC eyes, with a significant higher Sup − Inf value indicating asymmetry compared to the control group, with significantly higher MAX epithelial thickness, superior epithelial thinning, decreased MIN epithelial thickness, and more –ve values for MIN–MAX parameter (*p* = 0.004, 0.025, < 0.001, and < 0.001, respectively).

On the other hand, epithelial changes were less evident in palpebral-type VKC eyes as compared to the control group with the only significant changes that were noticed with a decrease in minimal epithelial thickness (*p* = 0.001).

Thus, we presume that the clinical presentation of the disease may be considered as an important predictor for the disease impact on corneal epithelium.

Further analysis to emphasize our hypothesis that the disease morphological clinical presentation might be considered as a predictor for the disease influence on the corneal epithelium,  the epithelial thickness map parameters studying in both subgroups, i.e., mixed and palpebral VKC, with the recorded significant differences between subgroups regarding the MIN epithelial thickness, MAX epithelial thickness and MIN–MAX values (*p* = 0.002, 0.008 and < 0.001, respectively)., and the observation that more epithelial changes were reported among the mixed type VKC eyes as compared to the palpebral type VKC eyes, again enhanced our suggestion that "the clinical presentation of the same disease may have an impact on how the cornea could be affected." Although the mean total central corneal thickness and minimum total thickness as displayed at the pachymetry maps revealed no statistically significant difference between mixed and palpebral subgroups, further analysis of the stromal thickness at the areas underlying the MAX and MIN epithelial thickness revealed a more significant thinning of the stroma in mixed VKC eyes at areas underlying MAX epithelial thickness on epithelial maps as compared with palpebral VKC eyes (*p* > 0.04), that was in agreement with other studies [9, 10], denoting that corneal epithelial redistribution and proliferation might be encountered in some corneas with stromal irregularities to compensate for that thinning.

Despite this may be the first work studying the corneal epithelial thickness map in VKC patients, we think that the current study may have the limitation of small sample size studied as well as the cross-sectional study design. A larger sample size as well as a long period of follow-up may be recommended to analyze the recorded corneal epithelial changes and their pattern of behavior. As these changes in epithelial thickness values are not specific for VKC disease, therefore, we recommend more studies to compare different patterns of corneal epithelial thickness mapping between different types of ocular surface disorders.

We recommend also studying the effect of long-term use of topical corticosteroid eye drops on corneal epithelial thickness mapping as no previous studies were done in that important point, as we believe that it may influence the epithelial map parameters in VKC eyes as well as the disease entity.
